# Zinc-induced protective effect for testicular ischemia-reperfusion injury by promoting antioxidation via microRNA-101-3p/Nrf2 pathway

**DOI:** 10.18632/aging.102348

**Published:** 2019-11-05

**Authors:** Zhiqiang Qin, Kai Zhu, Jianxin Xue, Pu Cao, Luwei Xu, Zheng Xu, Kai Liang, Jiageng Zhu, Ruipeng Jia

**Affiliations:** 1 Department of Urology, Nanjing First Hospital, Nanjing Medical University, Nanjing 210006, China; 2 Department of Urology, The First Affiliated Hospital of Nanjing Medical University, Nanjing 210029, China; 3 Department of Urology, The Second Hospital of Nanjing, Nanjing University of Chinese Medicine, Nanjing 210003, China

**Keywords:** testicular ischemia-reperfusion injury, zinc, microRNA-101-3p, antioxidation

## Abstract

The present study was performed to determine the protective effect of Zinc on the rat testicular ischemia-reperfusion (I/R) injury and its mechanism. *In vivo*, the pathological changes and the apoptosis index were significantly relieved in the rats with Low-dose Zinc pretreatment, compared to the I/R group. After Low-dose Zinc treatment, the levels of tissue Malondialdehyde (MDA) were significantly decreased, while tissue antioxidant indices were significantly increased. Meanwhile, the level of NF-κB was significantly lower compared to I/R group, while the levels of Nrf2-dependent antioxidant enzymes were significantly higher in Low-dose Zinc+I/R group. *In vitro*, Low-dose Zinc markedly increased Leydig cell (TM3) cell viability, and relieved testicular oxidative damage via down-regulating ROS. A total of 22 differently expressed microRNAs were screened out using microRNA microarray in rat testicular tissue caused by I/R injury, especially showing that miR-101-3p was selected as the target miRNA. Furthermore, the levels of Nrf2 and NF-κB were apparently increased/decreased in TM3 cells treated with Hypoxic/Reoxygenation (H/R) after miR-101-3p mimics/inhibitor. In addition, H/R-induced testicular oxidative damage was recovered in TM3 administrated with miR-101-3p inhibitor and si-Nrf2. Therefore, this study provided a novel insight for investigating protective effect of Zinc on testicular I/R injury by promoting antioxidation via miR-101-3p/Nrf2.

## INTRODUCTION

Testicular torsion is a common urologic emergency resulting from the rotation of the vascular pedicle of the testis, which leads to necrosis, loss of spermatogenesis and testicular damage, and even inevitable orchiectomy [1, 2]. Prompt diagnosis and emergency surgical intervention of testicular torsion are both important in prevention of the ischemic testis injury, consequent subfertility and infertility [3]. Conservative surgical management, including detorsion of the involved tissues and restoration of testicular blood flow, might provide early treatment. However, de-rotation mainly causes testicular ischemia-reperfusion (I/R) injury, which is hard to avoid. Therefore, it is an urgent priority to find an effective treatment to relieve I/R injury.

Oxygen supply to the testes reduced in the process of testicular torsion, and testicular detorsion also leads to the formation of nitrogen, reactive oxygen metabolites and inflammatory response, resulting in the overproduction of reactive oxygen species (ROS) [4, 5]. Previous studies have revealed that ROS overproduction, considered as one of the main possible causes of testicular I/R injury, could activate oxidizing enzymes and inflammatory response, subsequently resulting in cell membrane, cytoskeletal and mitochondrial damages [6–8]. Under oxidative stress conditions, the activation of nuclear transcription factor erythroid 2-related factor 2 (Nrf2) plays a leading role [9, 10]. It has also been proven that Nrf2 is an essential cytoprotective regulator considering genes encoding the expression of proteins of antioxidants, detoxification enzymes, and other mediators of stress response [11]. These genes confer the resistance to oxidative stress in various disorders, including catalase (CAT), superoxide dismutase (SOD), Glutathione S-transferase (GST), NADPH quinine oxidoreductase-1 (NQO1) and heme oxygenase-1 (HO-1) [12, 13]. In addition, nuclear factor kappa B (NF-κB), which can be activated by ROS in I/R injury, is one of the major redox-sensitive transcription factors controlling the expression of pro-inflammatory genes [14]. Pharmacological agents promoted the activation of Nrf2 and attenuated oxidative stress in I/R injury models, thus playing an important role in antioxidation effects [15].

Numerous free radical scavengers, antioxidants, anti-inflammatory cytokines, and other drugs have been investigated in testicular torsion/detorsion models and been shown preventing testicular I/R injury to mitigate adverse effects [7, 8]. Zinc is one essential bio-element and an important intracellular signaling mediator, which plays a fundamental role in a wide-ranging biochemical processes [16–19]. A recent report has also demonstrated that acute Zinc administration could partially restored antioxidant activity inhibited by oxidative stress in cardiac I/R in rats, and chronic Zinc supplementation could markedly elevate it [20]. Thus, induction of Nrf2 pathway might be considered as a potent protective tactics. However, it is still unknown whether Zinc administers to the protection of testicular I/R injury via Nrf2/HO-1 pathway.

MicroRNA (miRNA), which belongs to the small non-coding RNAs (ncRNAs), is able to regulate gene expression by binding with target mRNA [21]. The growing studies demonstrated that miRNAs played great roles in the progression of various diseases, suggesting that miRNAs might be considered as a potential drug target for the treatment of human diseases [19, 22–24]. Although the nature of the protective mechanisms underlying testicular ischemia by the addition of Zinc are not well understood, several studies have suggested that Zinc reduced I/R injury in various organs, such as the heart, kidney and other organs, via antioxidative effects [25, 26]. However, few studies have focused on the role of Low-dose Zinc on testicular I/R injury via miRNA and Nrf2 signaling pathway. Therefore, our study aimed to investigate the protective effect of Zinc on testicular I/R injury and to clarify the potential mechanism by which Zinc modulates miRNA/Nrf2 pathway and subsequent oxidative stress responses involved in the progression of testicular I/R injury.

## RESULTS

### Zinc prevented testicular ischemia-reperfusion (I/R) injury in rats

**Figure 1:**
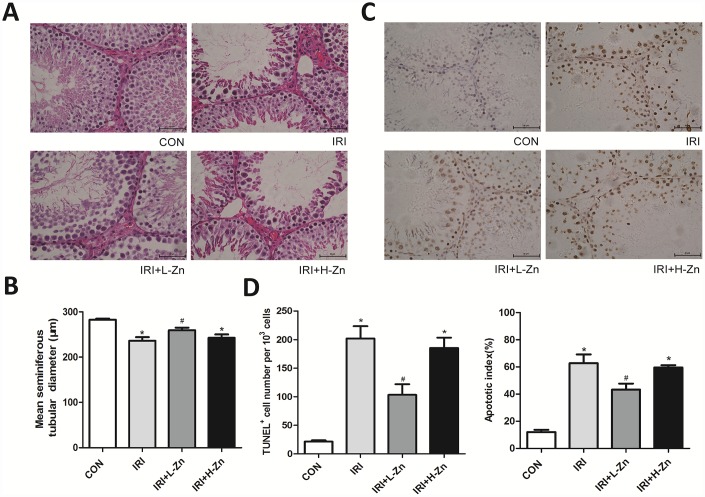
**Zinc prevented testicular ischemia-reperfusion (I/R) injury in rats.** (**A**) H&E staining of testicular tissues in Control, I/R injury, and I/R injury treated with Zinc rats (×400). Among them, ① Control group (n=8), ② I/R group (n=8), ③ Low-dose Zinc+I/R group (n=8), ④ High-dose Zinc group+I/R (n=8). (**B**) Mean seminiferous tubular diameter (MSTD) in each group. (**C**) TUNEL assay of testicular tissues in each group. Apoptotic cells exhibit a brown nuclear stain under microscope (×400). (**D**) TUNEL positive cells per 10^3^ germ cells and Apoptotic index of testes in each group. Data are shown as mean ± SD. *significant difference vs. Control group (*P< 0.05*); ^#^significant difference vs. I/R group (*P*< 0.05).

As shown in Figure 1A, the histopathological examination in I/R group showed congestion hemorrhage, germ cell loss, and disruption of the seminiferous epithelium, compared with Control group. In High-dose Zinc+I/R group, the pathological changes did not improve compared with I/R group. In contrast, the testicular tissues in Low-dose Zinc+I/R group showed an improved histological appearance compared to I/R group. As presented in Figure 1B, the MSTD in I/R group and High-dose Zinc+I/R group were significantly reduced compared to the values in Control group. In contrast, the MSTD in Low-dose Zinc+I/R group was significantly higher compared with I/R group (*P*<0.05). In addition, as shown in Figure 1C, compared with Control group, the number of testicular apoptotic cells in I/R group was significantly higher; Nevertheless, the percentage of apoptotic cells in Low-dose Zinc+I/R group was significantly lower than that of I/R group (*P*< 0.05), which provided evidence that Low-dose Zinc could protect against testicular I/R-induced apoptotic damage. Moreover, semi-quantitative analysis by both total TUNEL-positive cells/10^3^ germ cells and apoptotic index (AI) showed that Zinc could significantly decrease the incidence of testicular apoptosis and I/R-induced TUNEL-positive cells and AI (Figure 1D).

### Zinc alleviated testicular oxidative damage in rats

**Figure 2:**
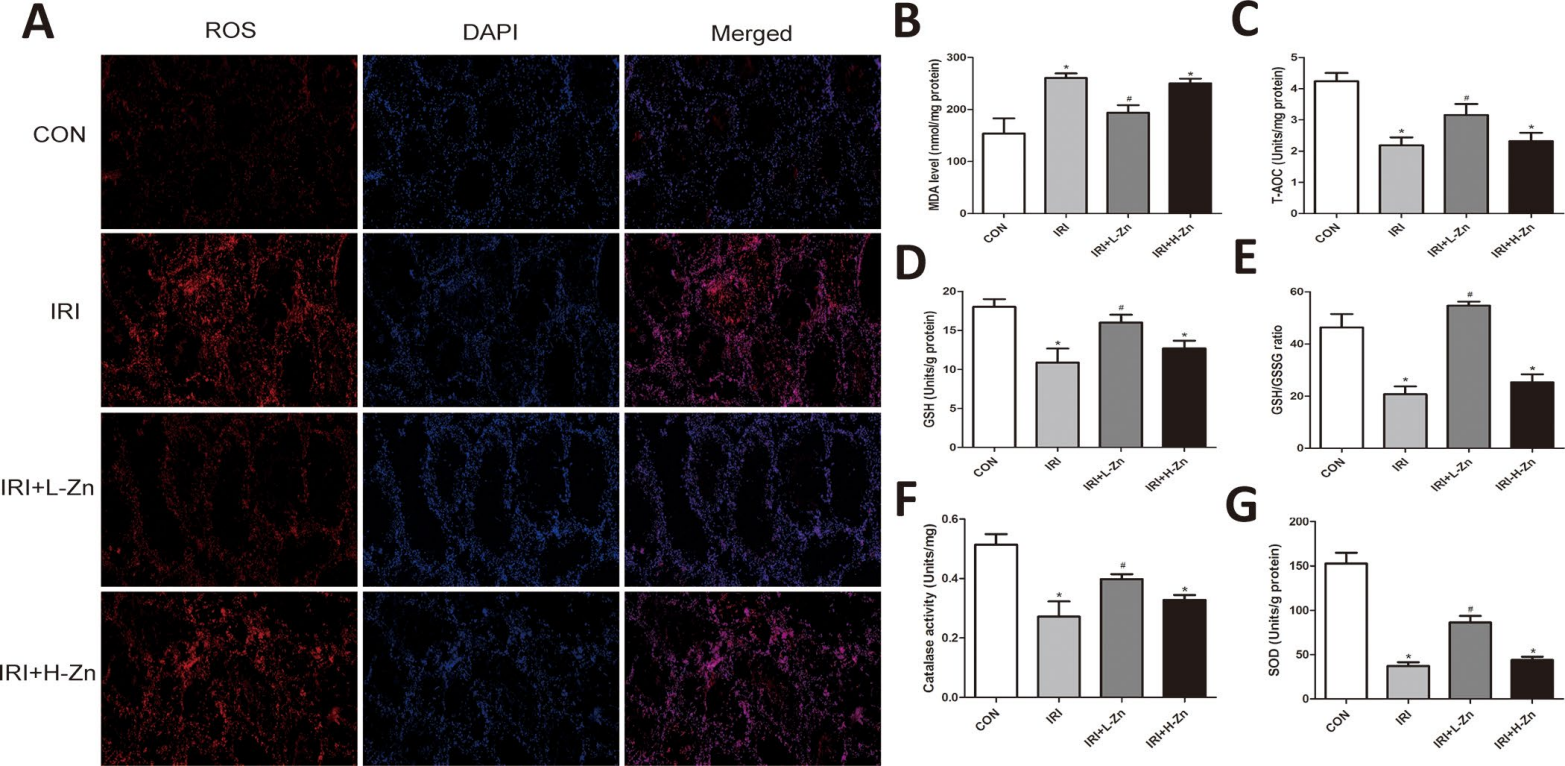
**Zinc alleviated I/R induced oxidative damage in testis tissues.** (**A**) DHE staining of testicular tissues in Control, I/R injury, and I/R injury treated with Zinc group. ROS exhibited red fluorescence under fluorescent microscope. (**B**–**G**) Content of MDA, T-AOC, GSH, GSH/GSSG, GST and SOD of testicular tissues in each group. Data are expressed as mean±SD. *significant difference vs. Control group (*P< 0.05*); ^#^significant difference vs. I/R group (*P< 0.05*).

The intensity of the dihydroethidium (DHE) staining of testis in Control group was low, while it was markedly enhanced in I/R group. In contrast, the administration of Low-dose Zinc +I/R significantly reduced the intensity of the fluorescent signals, indicating that it could reduce testicular oxidative stress compared to I/R group (Figure 2A). As shown in Figure 2B–2G, the MDA content in I/R and High-dose Zinc+I/R groups were significantly higher compared to Control group (*P*<0.05); However, compared to I/R group, Low-dose Zinc+I/R group could significantly rescue MDA level (*P*<0.05). In addition, Low-dose Zinc+I/R group could increase the level of antioxidant enzymes SOD and CAT, and the content of antioxidants T-AOC and GSH, GSH/GSSG to reduce testicular oxidative stress injury compared to I/R group. Nevertheless, these parameters in I/R group and High-dose Zinc+I/R group were significantly lower compared to Control group, which indicated that Low-dose Zinc alleviated testicular oxidative damage *in vivo*.

### Zinc pre-treatment increased the expression levels of Nrf2 and its downstream target genes in rats testicular I/R injury

**Figure 3:**
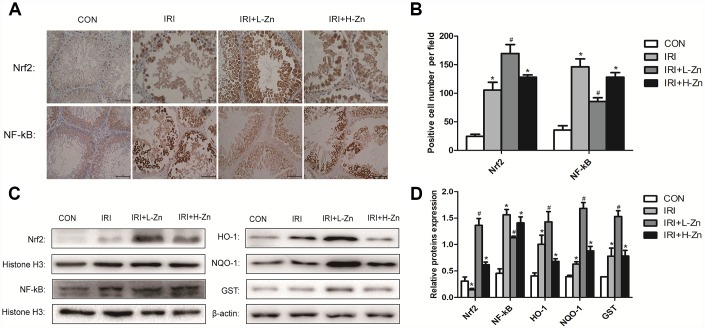
**Zinc pre-treatment increased the expression levels of Nrf2 and its downstream target genes in testicular tissues.** (**A**) Immunohistochemical Staining showed Nrf2 and NF-κB expression levels in Control, I/R injury and I/R injury treated with Zinc groups. (**B**) Statistical analyses of IHC results in Nrf2 and NF-κB expression levels in different groups. (**C**) Protein levels of Nrf2, NF-κB, HO-1, NQO-1 and GST in different groups. Histone H3 and β-actin were used as a protein control to normalize volume of protein expression. (**D**) Statistical analyses of Western Blotting results in Nrf2, NF-κB, HO-1, NQO-1 and GST protein levels in different groups. Data are expressed as mean± SD. *significant difference vs. Control group (*P*< 0.05); ^#^significant difference vs. I/R group (*P< 0.05*).

The expression levels of Nrf2 and NF-κB in the testes as assessed from immunohistochemical analysis were as follows. Compared to Control group, higher level of NF-κB expression were detected in I/R group (*P*<0.05); Nevertheless, the expression level of NF-κB was decreased in Low-dose Zinc+I/R group, compared to I/R group (*P*<0.05). In addition, the increased Nrf2 expression was significantly observed in Low-dose Zinc+I/R group compared to I/R group (*P*<0.05) (Figure 3A–3B). As shown in Figure 3C, 3D, the protein levels of Nrf2, NF-κB, HO-1, NQO1 and GST were analyzed by Western blotting. The expression levels of Nrf2 and its downstream target genes in Low-dose Zinc+I/R group were significantly increased compared with I/R group (*P*<0.05). As expected, NF-kB expression level in Low-dose Zinc+I/R group significantly decreased compared to I/R group (*P*<0.05).

### Differentially expressed miRNAs in testicular tissues of rats induced by I/R injury based on microarrays

**Figure 4:**
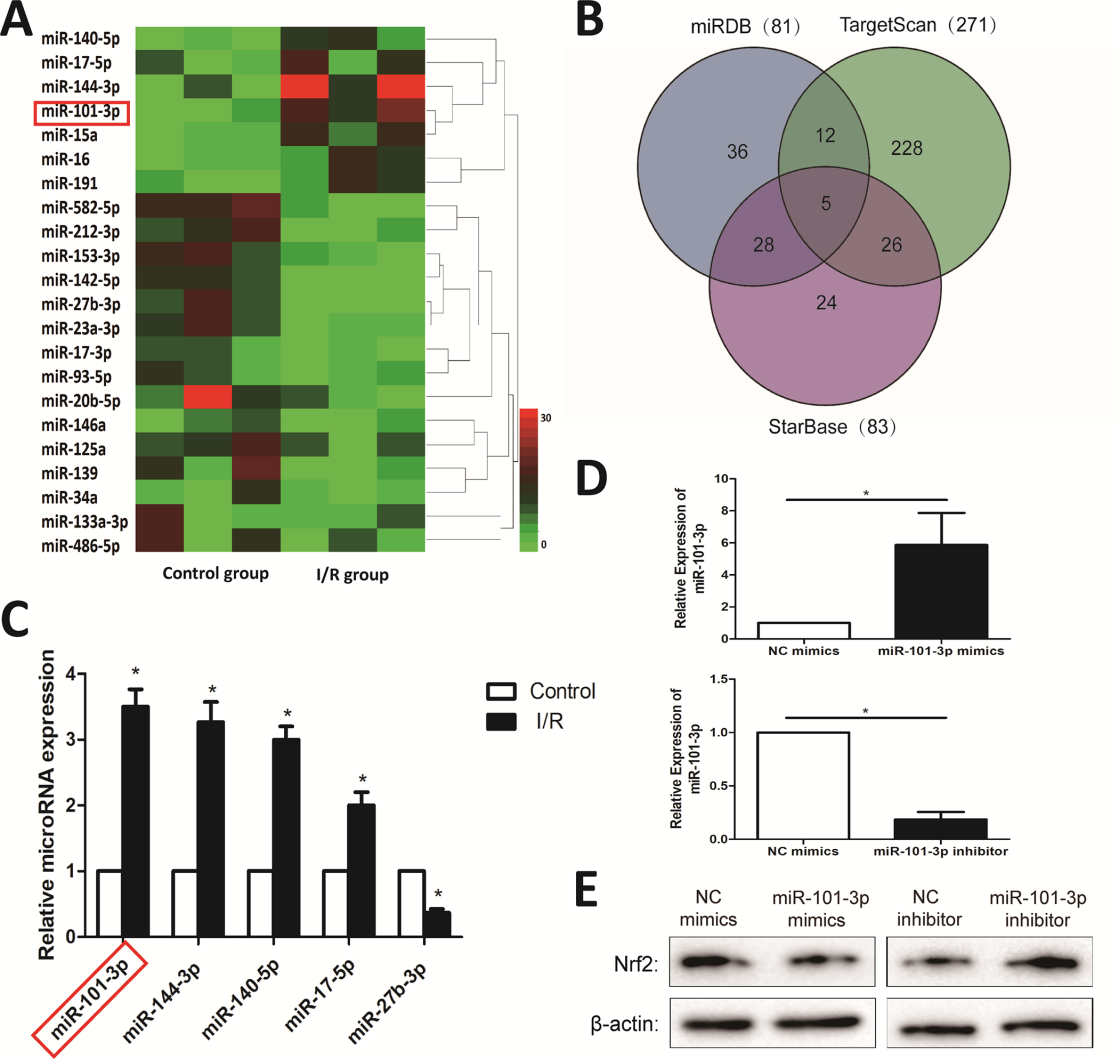
**Differentially expressed miRNAs in testicular tissues of rats induced by I/R injury based on microarrays.** (**A**) Hierarchical clustering analysis of the differentially expressed miRNAs between Control and I/R injury testis samples in rats. (**B**) A schematic diagram used to search the target miRNAs of Nrf2 in three databases. (**C**) Validation of the five differently expressed miRNAs in rats caused by I/R injury based on real-time PCR assay. (**D**) MiRNA-101-3p expression level of TM3 after transfecting miR-101-3p mimics and inhibitor. (**E**) Nrf2 expression level of TM3 after transfecting miR-101-3p mimics and inhibitor. All data are expressed as the mean ± SD. *significant difference vs. Control group (*P< 0.05*).

A total of 22 differentially expressed miRNAs with at least 2-fold changes and *P*-values less than 0.05 were identified in testicular tissues of rats caused by I/R injury using microRNA microarray analysis, compared with Control group. As shown in Figure 4A, the heat map indicated the results of a two-way hierarchical clustering of the samples and the 22 differentially expressed miRNAs, which displayed the relative expression levels identified by microarray. As shown in Figure 4B, the interaction networks between the Nrf2 and its upstream miRNAs were predicted by “miRDB”, “TargetScan” and “StarBase” databases. Then, 4 up-regulated and 1 down-regulated miRNAs were further validated by real-time PCR assay. As shown in Figure 4C, I/R injury significantly increased the expression levels of miR-101-3p, miR-144-3p, miR-140-5p, miR-17-5p, and markedly decreased the levels of miR-27b-3p in testicular tissues of rats compared with Control group. In addition, to investigate the the association between miR-101-3p and Nrf2 in TM3, miR-101-3p mimics and inhibitor was transfected into TM3. As shown in Figure 4D, miR-101-3p mimics significantly increased miR-101-3p in TM3, and miR-101-3p in TM3 was significantly decreased after administrated with miR-101-3p inhibitor. Then, Western Blotting showed that the protein level of Nrf2 decreased after administrated with miR-101-3p mimics; Nrf2 expression level increased after administrated with miR-101-3p inhibitor (Figure 4E).

### Low-dose Zinc up-regulated Nrf2 signaling pathway via miR-101-3p in TM3 Hypoxia/Reoxygenation (H/R) model

**Figure 5:**
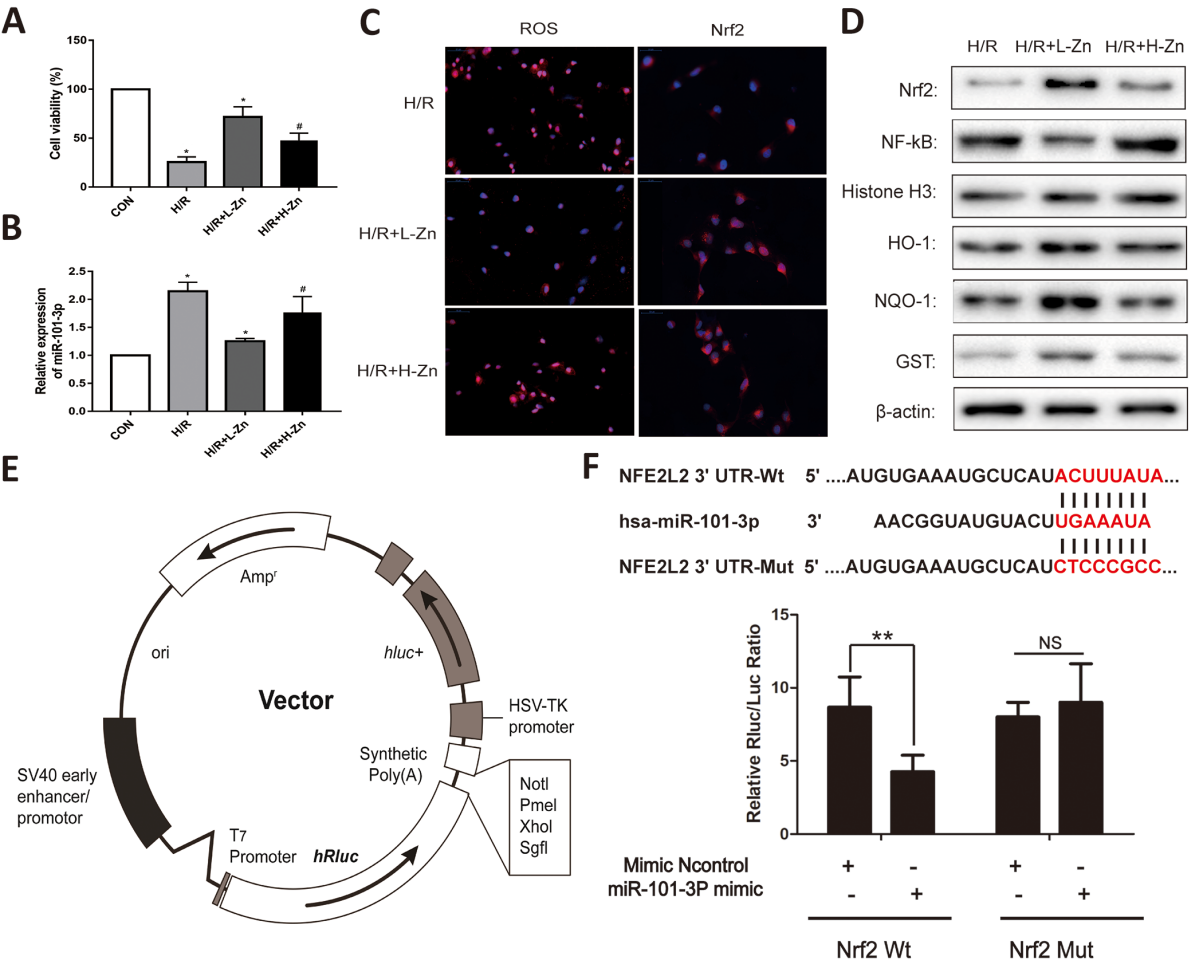
**Zinc up-regulated miR-101-3p via Nrf2/HO-1 signaling pathways in TM3 Hypoxia/Reoxygenation (H/R) model.** (**A**) Cell viability of TM3 in Control, H/R injury, and H/R injury treated with Zinc groups. (**B**) MiRNA-101-3p expression level in H/R with or without Zinc pretreatment groups. (**C**) The intracellular ROS and Nrf2 levels in H/R with or without Zinc pretreatment groups. (**D**) Protein levels of Nrf2, NF-κB, HO-1, NQO-1 and GST of TM3 in different groups. Histone H3 and β-actin were used as a protein control to normalize volume of protein expression. (**E**) The construction diagram of the target genes (Nrf2) double-luciferase reporter genes. (**F**) The relative luciferase expression with Nrf2 3′-UTR after co-transfection with miR-101-3p mimics or NC mimics in TM3. Data are expressed as mean±SD. *significant difference vs. Control group (*P< 0.05*); ^#^significant difference vs. H/R group (*P< 0.05*); **p < 0.01 compared with NC group; ns indicated no significance.

As shown in Figure 5A, the cell viability was evaluated when combined with or without H/R. The results found that the cell viability was significantly decreased in H/R group. When TM3 were co-culture with Zinc, the cell viability was relieved, and Low-dose Zinc+H/R group was markedly better than High-dose Zinc+H/R group. Subsequently, the results of real-time PCR assay indicated that the expression levels of miR-101-3p was significantly down-regulated in TM3 H/R model (*P*<0.05); Meanwhile, compared to High-dose Zinc+H/R group, Low-dose Zinc pretreatment could markedly decrease the expression level of miR-101-3p (*P*<0.05) (Figure 5B). As shown in Figure 5C, the intracellular ROS levels in Zinc pretreatment group was significantly lower than H/R group based on immunofluorescence assays. In contrast, Low-dose Zinc pretreatment could markedly decrease the excessive generation of ROS compared to High-dose Zinc+H/R group. In addition, the intracellular Nrf2 in Zinc pretreatment group was also significantly higher than H/R group, and Low-dose Zinc pretreatment could markedly increase Nrf2 compared to High-dose Zinc+H/R group. Then, the protein levels of Nrf2, NF-κB and its downstream signaling molecules were assessed to observe Zinc-induced protective effect for TM3 H/R model. As shown in Figure 5D, the levels of Nrf2, HO-1, NQO1 and GST were markedly increased, and NF-κB were significantly decreased by Zinc pretreatment compared with H/R groups *in vivo*. Moreover, Low-dose Zinc pretreatment could markedly improve the above markers compared to the High-dose Zinc+H/R group, which indicated that Low-dose Zinc could activate Nrf2/HO-1 signaling pathways to protect TM3 from hypoxic damage.

The RNA sequence alignment showed that the 3′-UTR of Nrf2 mRNA contained a complementary site for the seed region of miR-101-3p (Figure 5E). As presented in Figure 5F, the dual-luciferase reporter plasmid was obtained to perform the dual-luciferase reporter assay. In the groups of Nrf2-Wt, the luciferase activities were significantly repressed by miR-101-3p over-expression compared with mimics Control group. However, these effects were not observed with the mutated Nrf2 groups, suggesting that Nrf2 were the target genes of miR-101-3p.

### Transfecting miR-101-3p mimics and inhibitor regulated H/R-induced oxidative damage in vitro

**Figure 6:**
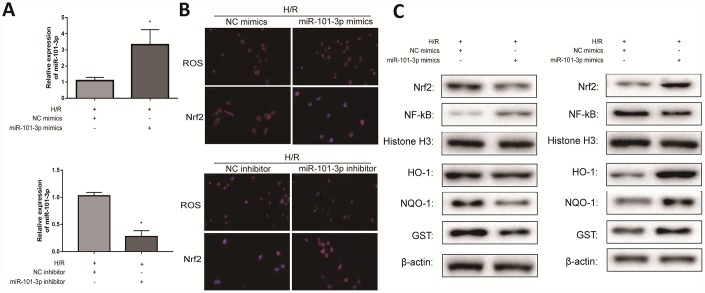
**Transfecting miR-101-3p mimics and inhibitor regulated H/R-induced oxidative damage *in vitro*.** (**A**) MiRNA-101-3p expression level of TM3 in H/R group after transfecting miR-101-3p mimics and inhibitor. (**B**) The intracellular ROS and Nrf2 of TM3 in H/R group after transfecting miR-101-3p mimics and inhibitor. (**C**) Protein levels of Nrf2, NF-κB, HO-1, NQO-1 and GST of TM3 after transfecting miR-101-3p mimics and inhibitor. Histone H3 and β-actin were used as a protein control to normalize volume of protein expression. Data are expressed as mean± SD. *significant difference vs. Control group (*P*< 0.05).

To investigate the role of miR-101-3p in TM3 H/R-induced oxidative damage, miR-101-3p mimics and inhibitor was transfected to TM3 in H/R model. As shown in Figure 6A, miR-101-3p mimics significantly increased miR-101-3p in TM3 H/R model, and miR-101-3p in TM3 H/R model was significantly decreased after administrated with miR-101-3p inhibitor. As presented in Figure 6B, the intracellular ROS in group transfected with miR-101-3p mimics was significantly higher than un-transfected group; However, the intracellular ROS in group transfected with miR-101-3p inhibitor was significantly lower than un-transfected group. In addition, the result of the intracellular Nrf2 was opposite after the same treatment. As shown in Figure 6C, the levels of Nrf2 and its downstream target genes HO-1, NQO1 and GST in TM3 H/R model were notably decreased and NF-κB was significantly increased after treating with miR-101-3p mimics compared with NC mimics group, and the results of protein levels were in contrast after treating with miR-101-3p inhibitor.

### H/R regulated Nrf2 signaling pathway through miR-101-3p in TM3

**Figure 7:**
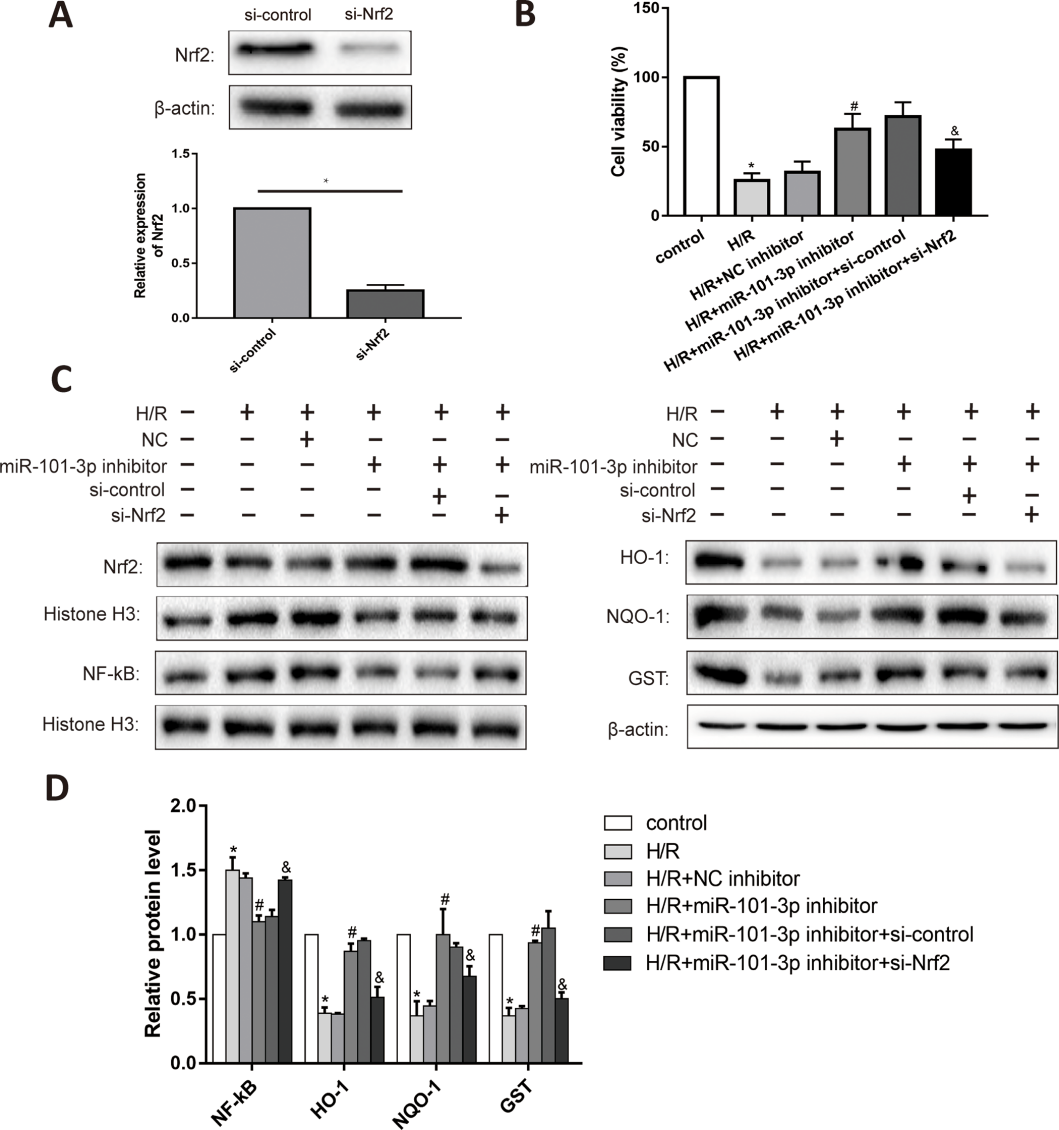
**The interaction between miR-101-3p and Nrf2 in TM3.** (**A**) Protein levels and statistical analyses of Western blotting results of Nrf2 in TM3 which administrated with si-Nrf2. (**B**) Cell viability of TM3 in different treated groups. (**C**) Protein levels of Nrf2, NF-κB, HO-1, NQO-1 and GST of TM3 in different groups. Histone H3 and β-actin were used as a protein control to normalize volume of protein expression. (**D**) Statistical analyses of Western blotting results in Nrf2, NF-κB, HO-1, NQO-1 and GST expression levels in different groups. Data are expressed as mean±SD. *significant difference vs. Control group (*P*< 0.05); ^#^significant difference vs. H/R+NC inhibitor+si-Control group (*P*< 0.05); ^&^significant difference vs. H/R+miR-101-3p inhibitor+si-Control group (*P*< 0.05).

As shown in Figure 7A, Nrf2 in TM3 cells was significantly decreased after administrated with si-Nrf2. Knockdown of miR-101-3p evaluated the cell viability in TM3 with H/R; Besides, si-Nrf2 further considerably reduced the effect of miR-101-3p inhibitor on the cell viability (Figure 7B). Knockdown of miR-101-3p via the transfection of miR-141-3p inhibitor reversed the H/R induced-promotion on NF-κB expression. Moreover, knockdown of miR-101-3p also reversed H/R induced-inhibition on the expression levels of Nrf2, HO-1, NQO1 and GST, while si-Nrf2 further considerably reduced the effect of miR-101-3p knockdown on Nrf2 and its downstream target genes (Figure 7C, 7D).

## DISCUSSION

To the best of our knowledge, the pathomechanism of I/R injury is multifactorial. Endothelial dysfunction and tubular cell injury through ATP depletion, accumulation of intracellular Ca^2+^, ROS and pro-inflammatory cytokines, and the apoptotic pathway have all been implicated [27, 28]. However, the increased production of oxygen-free radicals in conjunction with the decreased activity of antioxidant defenses is considered to be a significant factor for I/R injury [29].

Recently, many scientists have made great efforts to reduce I/R injury [25, 30, 31]. Among them, some evidences have suggested that Zinc plays a critical role in controlling I/R injury. Wang et al. reported that Zinc pretreatment might potentially preserve liver from heat-induced damage in mice, and was used for the purpose of additives for livestock feed at high temperatures [28]. In testicular I/R injury, however, the protective effect of Zinc has not been previously reported. Thus, we examined the protective mechanism of Zinc in testicular I/R injury via establishing research experimental model in adolescent male rats. Using histological and biochemical methods, as an effective assistance drug, Low-dose Zinc directly fought against I/R damage of the testes through its antioxidative properties. Consistent with preceding researches, we could easily find that High-dose Zinc didn’t improve histological appearance and decreased the number of apoptotic cells in Low-dose Zinc+I/R group, which meant the protective effect of Zinc on testicular I/R injury decreased with the increasing of Zinc during the dose range [31]. Meanwhile, the morphological change of testicular tissues in the High-dose Zinc+I/R group was close to those in I/R group; But it was significantly different from those in Low-dose Zinc+I/R group, indicating that Low-dose Zinc played a preventive effect on testicular I/R injury.

A growing number of studies further strengthened the evidences linking some biological pointers with oxidative stress [32–34]. ROS, chiefly oxygen free radicals, and MDA, as the end product of lipid peroxidation, involved in mediating oxidative damage during I/R injury, which have been documented extensively [35, 36]. Relatively, antioxidant defense systems, such as SOD, glutathione peroxidase (GSH-Px), CAT, and glutathione reductase (GSSGR) could protect organism from ROS [37]. In this study, we found that ROS and MDA significant increased in testicular I/R injury; But, Low-dose Zinc pre-treatment, as a significant reduction in ROS and an enhancement of antioxidant defense systems, could activate antioxidant pathway to relieve testicular I/R injury. Therefore, oxidative stress might be a critical factor that could aggravate I/R-induced testicular injury, and Low-dose Zinc had a protective effect on testicular I/R injury.

Nrf2 has been shown to be an important transcription factor induced by the antioxidant response [10]. Nrf2 could activate a battery of antioxidant genes, HO-1, NQO1 and other genes, which regulated oxidative stress, inflammatory reactions and apoptosis in various disorders [12, 38, 39]. Furthermore, NF-κB is also an important transcription factor that plays an important role in redox changes [14]. Zinc, known as an Nrf2 inducer, has been proven as an activator of the expression of Nrf2-mediated gene and inhibited cell apoptosis against renal I/R injury [26]. Moreover, Zinc down-regulated the activity of NADPH oxidase and directly neutralized ROS, thereby reducing ROS concentration. In addition, a decrease in ROS concentration inhibited NF-κB activation. Zinc could inhibit the phosphorylation of the inhibitory subunit of NF-κB complex to prevent the activation of NF-κB by reducing the production of ROS [26]. In the present study, compared to I/R group, the expression of Nrf2 and its downstream target genes were increased in the Low-dose Zinc+I/R group. Nevertheless, the anti-inflammatory effects of Low-dose Zinc in testicular I/R injury could inhibit NF-κB activation via a variety of mechanisms. Taken together, these data confirmed that Low-dose Zinc from the reaction between free radicals and anti-inflammatory enhanced the activation of Nrf2 and inhibited the expression of NF-κB in testicular I/R injury.

More and more studies have proved that oxidative stress could be regulated by multiple molecular mechanisms [38–40]. As research progresses, microRNAs has been shown to be one of the potential targets for the treatment of human diseases [41–43]. In the present study, we used microRNA microarray to screen out a total of 22 differentially expressed microRNAs in testicular tissues of rats caused by I/R injury, and miR-101-3p was aberrantly up-regulated in TM3 H/R model, as well as the testicular tissues induced by I/R injury. MiR-101-3p has also been confirmed to play important roles in inflammatory disease [41, 44]. However, no research to report the effects of miR-101-3p on Zinc-induced protective effect for testicular I/R injury was found, and thus miR-101-3p might be considered as one potential therapeutic target to relieve testicular I/R injury. And meanwhile, based on the bioinformatics database and double-luciferase reporter assay, the results showed that miR-101-3p could directly target Nrf2. Thus, miR-101-3p might be through adjusting Nrf2 signal pathways to resist Zinc-induced protective effect. In this study, miR-101-3p significantly down-regulated the expression level of Nrf2 and up-regulated the level of NF-κB, then reduced the expression levels of GST, NQO1 and HO-1, which thereby miR-101-3p might be a damage factor in testicular I/R injury. Furthermore, the intracellular ROS was apparently increased or decreased in TM3 H/R model administrated with Zinc after miR-101-3p mimics or miR-101-3p inhibitor transfection, as well as the expression change of Nrf2 signals. As the change of histopathological and the expression levels of Nrf2 and NF-κB, the proteins associated Nrf2 signal pathways were also affected. Thus, the above results demonstrated that miR-101-3p directly targeted Nrf2, and Zinc could down-regulated miR-101-3p to activate Nrf2 signaling pathways to relieve testicular I/R injury in rats.

To a certain extent, some limitations should also be emphasized when interpreting the data. (1) The 14-day treatment of Low-dose Zinc before the torsion injury could not be applied in clinical setting, since testicular torsion is unpredictable and not warranting preventive strategies. (2) The effect of acute Low-dose Zinc treatment after testicular torsion should be considered in the study, to understand the difference of antioxidant drug use before and after de-rotation operation. (3) The pre-treatment of Low-dose Zinc on testicular I/R injury was neither widely practiced nor supported by clinical studies. Thus, further exploration in the effects of Low-dose Zinc on testicular I/R injury might be performed to obtain more accuracy results in subsequent years.

## CONCLUSIONS

To summarize, we demonstrated that exogenous supplement of Low-dose Zinc showed a protective effect on testicular I/R injury, and it potentially down-regulated miR-101-3p to promote its antioxidative effects via targeting Nrf2. Moreover, further studies are needed to recapitulate such results using different measurement methods.

## MATERIALS AND METHODS

### Animals and experimental protocol

A total of 32 adolescent male Sprague-Dawley (SD) rats (180–220 g) at the age of averaging 6 weeks old were purchased from the Animal Experiment Center of Nanjing Medical University (Nanjing, Jiangsu, China). These rats were randomly divided into four groups: Control group (n=8), I/R group (n=8), High-dose Zinc+I/R group (60 mg/kg body weight) (n=8), and Low-dose Zinc+I/R group (15 mg/kg body weight) (n=8). The testes were examined via a scrotal incision and then replaced in the scrotum without torsion in Control group. In I/R group, the testes were rotated 720° in the clockwise direction for two hours, and then testicular reperfusion was performed by restoring the testes to their normal position. Subsequently, orchiectomy was performed four hours later. In High-dose Zinc+I/R group and Low-dose Zinc+I/R group, the same testicular torsion process was performed with Zinc (Santa Cruz, CA, USA) pre-treated for 14 consecutive days prior to testicular I/R injury surgery. The same volume of diluted solution was gavaged in Control and I/R groups. On the day of sacrifice, overnight fasted rats were anaesthetized with intraperitoneal injection of Pentobarbital (40 mg/kg BW).

All procedures conducted in experimental animals and the protocols were approved by the Committee on the Ethics of Animal Research in Animal Care Facility of Nanjing Medical University (Nanjing, Jiangsu, China). Unnecessary pain or stress was avoided and animal manipulation was performed with maximal care and hygiene. The study was carried out in strict accordance with the recommendations in the Guide for the Care and Use of Laboratory Animals of the National Institutes of Health.

### Surgical procedure

Surgical procedures were carried out by sterile techniques and performed under chloral hydrate anesthesia (300 mg/kg, intraperitoneally). Incising the ilioinguinal and exposing the testicle and spermatic cord. The left testis was rotated 720° in the clockwise direction for torsion in I/R group and Zinc-treated groups. To ensure this torsion position, we fixed the tunica albuginea to the scrotum with a 5/0 silk suture. Detorsion was operated by restoring the testes to their normal position after 2 hours of ischemia. Subsequently, orchiectomy was performed 4 hours later. The same surgical procedure was performed at the right testis on each rat. At the end, we collected testis tissues for Histopathological examination, Biochemical analysis, and Western blotting.

### Histological examination

Testicular tissue samples were fixed in 10% formaldehyde for 24 hours, dehydrated, embedded in paraffin, sectioned at 5 μm, and stained with hematoxylin and eosin. Next, the sections were evaluated under a standard light microscopy (Olympus BX-51, Tokyo, Japan) for the change of testis structural by two blinded investigators. The mean seminiferous tubular diameter (MSTD) was measured in the same histological section on 5 different focuses with a microscope-adaptable micrometer.

### TdT-mediated dUTP nick-end labeling (TUNEL) assay

TUNEL-staining assays were performed according to the manufacturer’s instructions (In Situ Apoptosis Detection Kit, Roche, Basel, Switzerland). The nucleus of any apoptotic cell would exhibit a brown stain under a standard fluorescence microscope, and this was manually analyzed in a blinded fashion. TUNEL-positive cells was presented per 10^3^ germ cells. On each slide, the apoptotic index was calculated by quantifying the number of TUNEL-positive versus total cell nuclei from five randomly chosen high-power fields (400×) containing 10^3^ cells.

### Biochemical measurements

The level of Malondialdehyde (MDA), CAT, total antioxidant capacity (T-AOC), glutathione (GSH), SOD, and glutathione/glutathione disulfide ratio (GSH/GSSG) were determined by using an Assay Kit (Jiancheng Bioengineering Institute, Nanjing, China) [19, 23]. According to the method described by Benov et al. [24], we evaluated testicular intracellular ROS production by performing the intracellular superoxide assay under a standard fluorescence microscope (Eclipse Ti-SR, Nikon Co, Japan) and detected the density of the images by using a standard fluorescence spectrophotometer (arbitrary units per millimeter square field).

### Immunohistochemical (IHC) staining

Immunohistochemistry was performed on paraffin testicle sections at 5 μm of thickness. For specific staining, we incubated slides with rabbit anti-mouse Nrf2 and NF-κB specific antibody (Cell Signaling Technology, USA) overnight at 4 °C. Washing and incubating the slides with a 1:100 dilution horseradish peroxidase (HRP)-conjugated anti-goat secondary antibody (Cell Signaling Technology, USA) for 1 hour the next day.

### Cell culture and transfection

The Leydig cell (TM3) was purchased from the Shanghai Institutes for Biological Sciences (Shanghai, China). TM3 was maintained in DMEM supplemented with 10% FBS and antibiotics (100 IU/mL penicillin and 100 mg/mL streptomycin) in a humidified atmosphere of 5% CO_2_ and 95% O_2_ at 37°C. Prepare 50 μmol/L and 200 μmol/L Zinc-containing medium as Low-dose Zinc medium and High-dose Zinc medium, respectively. Transfection was performed to up-regulate or down-regulate the expression levels of miR-101-3p. Briefly, dissolving the miR-101-3p mimics, Negative Control mimics, miR-101-3p inhibitor or Negative Control inhibitor in Opti-MEM separately. The solutions were equilibrated for 5 mins at room temperature. Then, according to the manufacturer's protocol, combining each solution with Lipofectamine 3000 transfection reagent and mixing the solution gently allowed to form inhibitor liposomes for 20 mins. TM3 was transfected with the transfection mixture in serum-free cell medium. Place the mixed in a humidified atmosphere of 5% CO_2_ and 95% O_2_ at 37°C. Six hours later, replace the cell medium with fresh Medium. Finally, the expression levels of miR-101-3p and target genes was detected.

### Cell hypoxic/reoxygenation (H/R) model

According to the manufacturer's protocol, cells were incubated under hypoxic conditions in a modular incubator chamber (Billumps-Rothenberg, Del Mar, CA). Place TM3 in the chamber flushed with a mixture of 94% N_2_, 5% CO_2_ and 1% O_2_ at 37°C for 24 hours (oxygen-serum deprivation, ischemic cells) [45]. Next, return duplicate hypoxic cultures for reoxygenation in the normoxic incubator and serum-containing medium for 6 hours, and serve TM3 incubated under normoxic conditions as Controls.

### Cell viability assay

According to the manufacturer's protocol, the cell viability with CCK-8 (Dojindo Laboratories, Kumamoto, Japan) was assessed. In brief, 10 μl CCK-8 solution was added to each plate and cells were incubated for 2 h at 37° after different treatments. Revealing the cell viability by the absorbance which was measured at 450 nm.

### miRNA microarray

According to the manufacturer's instructions, total RNA samples from I/R group and Control group of rats (n=5) was isolated using TRIzol reagent (Invitrogen, USA) and purified using RNeasy mini kit (Qiagen, Germany). The hierarchical clustering was performed to show distinguishable miRNA expression profiling among samples.

### Prediction of target miRNAs

The target miRNAs of Nrf2 validated above were screened by the “miRDB”, “TargetScan” and “StarBase” databases, and each target miRNA must be searched in at least two databases. Ultimately, the potential target gene was obtained.

### Dual-luciferase reporter assay

Establishing the plasmids containing the wild-type miR-101-3p-Nrf2 (wt-Luc-Nrf2) response element and the corresponding mutant (mut-Luc-Nrf2), the blank plasmids were purchased from RiboBio. Co., Ltd. (Guangzhou, China). Co-transfecting Plasmid DNA (wt-Luc-Nrf2, mut-Luc-Nrf2) and miR-101-3p mimics or NC mimics into TM3. Finally, the luciferase activity was assessed with a Double-Luciferase Reporter Assay Kit which purchased from Promega Biotech Co., Ltd. (Beijing, China) and the Dual-Light Chemiluminescent Reporter Gene Assay System (Berthold, Germany) was used to firefly luciferase activity.

### Immunofluorescence assay

Immunofluorescence staining in tissue sections or cells was performed using antibody in a humidified box at 4°C overnight, and followed by incubation with an Alexa fluorescein-labeled secondary antibody for 1 h at 37°C. The cell nuclei were stained with DAPI (5.0 μg/mL). Immunostained samples were imaged by fluorescence microscopy (Olympus, Japan) at 200× magnification.

### The detection of differentially expressed miRNAs and mRNAs

According to the manufacturer's protocol, total RNA samples from TM3 and testicular tissues of rats were obtained in different treatments using TransZol. The SYBR Premix Ex TaqII of SYBR PrimeScript™ miRNA RT-PCR kit (Takara, Dalian, China) was used to reverse transcribed synthesis of cDNA to evaluate the expression level of miRNAs. Use SYBR PrimeScript™ miRNA RT-PCR kit to complete the following steps of qRT-PCR. Then, the MMLV Reverse Transcriptase First Strand cDNA Synthesis Kit (Invitrogen, Carlsbad, USA) was used to synthesize cDNA to assess the mRNA levels. qRT-PCR with SYBR Green Real-time PCR kit (Takara, Dalian, China) was performed on Applied Biosystems 7900HT Sequence Detection System (United States). We selected U6 and β-Actin as internal control for normalization of miRNAs and mRNAs, and analyzed the relative levels of gene expression by 2^−ΔΔCt^ method. In addition, each experiment was asked to repeat three times. The following primers were used for qRT-PCR: Nrf2, forward 5′-CTTTAGTCAGCGAC AGAAGGAC-3′; reverse 5′-AGGCATCTTGTTTGGG AATGTG-3′ NF-κB, forward 5′-CCAACAGATGGCC CATACCT-3′; reverse 5′-AACCTTTGCTGGTCCCA CAT-3′ Histone H3, forward 5′-CAAGCGCGTCAC TATCATGC-3′; reverse 5′-CCTTTGCTTGGACCGT CAGA-3′ HO-1, forward 5′-AGGTACACATCCAAGC CGAGA-3′; reverse 5′-CATCACCAGCTTAAAGCC TTCT-3′ NQO1, forward 5′-AGGATGGGAGGTACTC GAATC-3′; reverse 5′-TGCTAGAGATGACTCGGAA GG-3′ GST, forward 5′-TGCTGTCTTCTCTTCTTC CCAG-3′; reverse 5′-CCCAAAGGCTCCGTATCTG C-3′ β-Actin, forward 5′-CCTGGCACCCAGCACA AT-3′; reverse 5′-GCTGATCCACATCTGCTGGAA-3′.

### Western blot analysis

According to the manufacturer's instructions, total protein was extracted from freshly obtained testicular tissues using a Nuclear Extract Kit (Active Motif, Tokyo, Japan). Primary antibodies against Nrf2, NF-κB, GST, HO-1 and NQO1 (Cell Signaling Technology, USA) were used in this study. We used Histone H3 and β-actin (Cell Signaling Technology, USA) as a protein control to normalize the volume of protein expression. The densitometric analysis of the protein bands was performed quantitatively with Image Lab Software (Bio-Rad, USA).

### Statistical analysis

The experimental results were statistically analyzed by SPSS 22.0 (Armonk, New York, USA). The measurement data were expressed as the mean ± standard deviation (SD) for each group. It was performed with analysis of variance (ANOVA) followed by Dunnett's test when comparing multiple groups to evaluate the significance of differences between groups. Statistical significance was considered to be *P* < 0.05.
